# Demand analysis of general practice patients for teaching clinic based on Kano model

**DOI:** 10.3389/fpubh.2024.1336683

**Published:** 2024-02-21

**Authors:** Zhiqian Chen, Ankang Yin, Yi Wang

**Affiliations:** ^1^General Practice, The Affiliated Hospital of Yangzhou University, Yangzhou, China; ^2^Graduate School of Dalian Medical University, Dalian, China

**Keywords:** general practice, teaching clinic, service demand, Kano model, medical education

## Abstract

**Background:**

General practice teaching clinics play a crucial role in the training of general practitioners, as they are more likely to enhance reception skills compared to traditional training methods. The quality of teaching clinics is largely determined by the level of patient acceptance. In recent years, the Kano model has become increasingly popular in the healthcare industry and has been used to enhance patient satisfaction. The objective of this study is to apply the Kano model to investigate the needs of patients in general practice teaching clinics and to rank the significance of each demand. This study will serve as a reference for enhancing the service quality of teaching clinics and advancing the field of general practice.

**Methods:**

A total of 101 patients of general practice at the Affiliated Hospital of Yangzhou University in Jiangsu province were selected using a random convenience sampling method to participate in a questionnaire survey. The questionnaire was designed by members of our team and was based on the Kano model. The study defined the service demand, assessed the impact of both satisfaction and dissatisfaction and created a matrix bubble diagram.

**Results:**

The study findings revealed that out of the 14 items of the general practice teaching clinic service demands, 1 item was categorized as a must-be requirement, 4 items were categorized as one-dimensional requirements, 2 items were categorized as an attractive requirement, 2 items were categorized as an indifferent requirement, and 5 items were categorized as mixed attributes. The findings of the matrix analysis showed that 4 items were situated in the area of one-dimensional attributes quadrant, 3 items were situated in the area of attractive attributes quadrant, 5 items were situated in the area of indifferent attributes quadrant, and 2 items were situated in the area of must-be attributes quadrant.

**Conclusion:**

The patients of general practice have positive attitudes toward teaching clinics. The findings can offer valuable insights for enhancing the quality of service and patient experience in general practice teaching clinics as well as for advancing the field of general practice.

## Introduction

Primary healthcare is the key to achieving universal health coverage and ensuring health for all. As the “gatekeeper” of citizens’ health needs, general practitioners play an important role in providing basic medical and health services (1). Accelerating the training of a large number of competent general practitioners is of great significance for strengthening the development of the community-level medical and health service system as well as preserving and enhancing public health. The general medicine teaching clinic is a kind of medical practice in which the resident doctor takes the lead in conducting medical history interviews, physical examinations, diagnoses, and treatments. This type of teaching is centered on the student and brings students to authentic clinical scenarios (2), which can effectively enhance the overall competency of resident physicians during their training.

At present, general medicine teaching demonstration bases nationwide are carrying out general medicine teaching clinics. A positive experience in clinical practice and the presence of role models are crucial for students (3). However, traditional training methods are based on observation, and students lack active communication and active thinking with patients (4). Teaching clinics effectively make up for the shortcomings of traditional training methods and help in improving students’ mastery of theoretical knowledge, initial diagnostic capabilities, doctor–patient communication skills, clinical operation, and the ability to work cohesively (5–7), Consequently, teaching clinics have emerged as a significant component of China’s medical education reform (8). Previous studies of teaching clinics have focused on resident physician trainees and lead trainers, with limited consideration given to the feelings and needs of patients in the teaching clinic. Nevertheless, patients are an important part of the teaching clinic (9, 10).

The Kano model is a typical qualitative analysis model that can be used to identify customers’ acceptance of new features, and it has been widely used in medical service investigation (11–13). This study aims to understand the needs of general medicine patients for teaching clinical services through the Kano model. It seeks to analyze the quality attributes of general medicine teaching clinic service programs and provide references to enhance the quality of general medicine teaching clinic service, improve patients’ access experience, and contribute to the advancement of general medicine.

## Participants and methods

### Participants

Patients in the general practice department of the Affiliated Hospital of Yangzhou University from June 2023 to July 2023 were randomly selected using the random sampling method. The inclusion criteria were as follows: (1) volunteered for the study; (2) aged 18 years old and above; (3) clear consciousness, normal cognitive function, and freedom of expression. The exclusion criteria were as follows: (1) participants with severe physical illness, obvious mental disorder, or cognitive impairment; (2) participants whose communication and presentation skills did not meet the requirements; (3) hospital staff. All participants could withdraw from the study at any time.

According to the Kendall sampling estimation method (14), the sample size of a study should be 5–10 times the number of independent variables. The number of independent variables in this study was 14. Assuming a loss to follow-up rate of no more than 20%, the sample size required for this study was (14*5)*1.2–(14*10)*1.2; that is, the sample size range was 84–168 cases. Ultimately, 101 cases were finally included in the study.

### Survey questionnaire

This study employed a questionnaire survey, which was administered to patients one by one by the investigator. A questionnaire for outpatient service needs based on the Kano model was designed to meet the real situation of the general medicine teaching clinic. The Kano model uses a questionnaire survey to distinguish the different needs of patients, not only in the form of a single choice but also in the two aspects of positive and negative questions. The alternative responses were as follows: (1) I like it that way, (2) It must be that way, (3) I am neutral, (4) I can live with it that way, (5) I dislike it that way; as shown in Table 1.

**Table 1:** Traditional Kano model categories.

Functional form of the question	Dysfunctional form of the question				
	I like it that way	It must be that way	I am neutral	I can live with it that way	I dislike it that way
I like it that way	Q	A	A	A	O
It must be that way	R	I	I	I	M
I am neutral	R	I	I	I	M
I can live with it that way	R	I	I	I	M
I dislike it that way	R	R	R	R	Q

According to the requirements of the Kano model, the forward and reverse answers of the questionnaire were classified by two-dimensional attributes. It classifies service quality attributes into one of the six categories of perceived quality, where M represents must-be attributes, O represents one-dimensional attributes, A represents attractive attributes, I represents indifferent attributes, R represents reverse attributes, and Q represents questionable attributes.

Cronbach’s alpha is a measure of the internal consistency of an instrument and is expressed as a number between 0 and 1. A Cronbach’s alpha value of 0.7 is generally considered to be an acceptable reliability coefficient as Materla et al. previously stated (13). Based on the above criteria, our preliminary experiment Kano questionnaire had acceptable internal consistency, with standardized Cronbach’s alpha values of 0.871 and 0.827 for forward and reverse questionnaires, respectively. Kaiser–Meyer–Olkin (KMO) measures and Bartlett’s test of sphericity were used to test the validity, and the KMO values for the forward and reverse questionnaires were 0.705 and 0.716, respectively, which were greater than 0.7, and Bartlett’s test of Sphericity values of *p* were 0.000, which were less than 0.005, which indicated that the questionnaires had good validity (15–17).

### Research method

The judgment of the mixed category is to confirm the traditional Kano category twice by calculating the Total Strength (TS) and Category Strength (CS) (18). The TS reflects whether the user is satisfied with a certain product or service theme, while the CS reflects the user’s recognition that the product or service theme belongs to a certain category, TA and CS are as follows: TS = (M + O + A)/(A + O + M + I + R + Q); CS = (max{A,O,M,I,R,Q}-second max {A,O,M,I,R,Q})/(A + O + M + I + R + Q). When TS is ≥60% and CS is ≤6%, the service belongs to the mixed category. The main components of the mixed category are the first two Kano categories with the highest proportion. Satisfaction Influence (SI) and Dissatisfied Influence (DSI) are used to determine how sensitive customers are to changes in the levels of attribute characteristics and thus to identify key improvement factors (19). The absolute values of the SI and DSI were between 0 and 1, the closer the SI is to 1, the greater the impact of the service on patient satisfaction. The closer the DSI is to −1, the greater the impact of the service on patient dissatisfaction. Values closer to 0 suggest that the service had very little effect on satisfaction or dissatisfaction. SI and DSI are as follows: SI = (A + O)/(A + O + M + I); DSI = (−1) × [(O + M)/(A + O + M + I)]. The matrix graph is drawn according to SI and DSI. The horizontal coordinate is the DSI value, and the vertical coordinate is the SI value. The matrix graph is restricted, and an importance matrix analysis is carried out to determine the priority of the improvement of each index.

### Statistical analysis

The data from the questionnaire were organized and imported into Excel tables. SPSS version 24.0 was used for conducting statistical analysis and reliability and validity tests. The general data of the research subjects are described by frequency or percentage. The attributes of service demand were judged according to the two-dimensional attribute categorization method of the Kano model. The SI and DSI of each service were calculated, and the importance of each service item was analyzed by making a matrix diagram according to it.

## Results

### General information on surveyed patients

In total, 120 questionnaires were collected, of which 101 were valid, and the effective recovery rate was 85.17%. Among the 101 participants, 46 were men, 55 were women, and their average age was 62.82 years. The data showed that 31.7% of people received a middle school education, 22.8% received a high school and junior college education, and 11.9% received a higher education (Table 2).

**Table 2:** Sociodemographic characteristics of the patients.

Sociodemographic characteristics	Frequency (%)
Gender	
Men	46 (45.5)
Women	55 (54.5)
Age (years)	
18–45	9 (8.9)
46–55	18 (17.8)
56–65	27 (26.7)
66–75	32 (31.7)
76 +	15 (14.9)
Education	
Elementary school and below	34 (33.7)
Middle school	32 (31.7)
High school and junior college	23 (22.8)
High education	12 (11.9)

### Kano attribute of demand of teaching clinic for patients of general practice

Collating the questionnaire data reveals that the frequency of each service item corresponds to its associated quality attribute, where the highest frequency is the corresponding attribute for that service item. Among the 14 service items, there were 5 items of one-dimensional attributes, 5 items of attractive attributes, 2 items of indifferent attributes, 2 items of must-be attributes, and no reverse attributes or questionable answers (Table 3).

**Table 3:** Kano attributes of demand of teaching clinics for patients of general practice.

Number	Service item	Frequency						Traditional category
		A	I	M	O	R	Q	
1	A comfortable clinic environment	31	17	30	23	0	0	A
2	Various convenient ways of registration	32	10	34	25	0	0	M
3	Advance notice of the specific process and time	35	16	26	24	0	0	A
4	Good service attitude	32	9	27	33	0	0	O
5	The resident physician in training leads the treatment process	39	44	5	6	7	0	I
6	The superior physician provides supplementary consultation	15	1	38	47	0	0	O
7	Participate in teacher–student interaction	37	44	3	10	7	0	I
8	Explain the condition and test results in detail and agree on the treatment plan together	30	29	21	21	0	0	A
9	Sufficient consultation time (30 min)	34	30	15	22	0	0	A
10	Health guidance on lifestyle and home care	44	12	12	33	0	0	A
11	Skilled in the inspection and operation	9	3	54	35	0	0	M
12	Fixed medical team and consistent treatment	14	28	24	35	0	0	O
13	Reasonable cost for examinations and treatments	5	5	38	53	0	0	O
14	Consistent efficacy meets expectations	2	2	31	66	0	0	O

### Category strength, total strength, and optimized Kano category of each general practice teaching clinic service item

The traditional Kano model categorization Method only considers the attribute category with the highest proportion, and generally does not consider other categories, which makes it difficult to meet the requirements of rigorous management when the second-highest value of the attribute frequency statistics is close to the highest value. Therefore, we categorize them again according to the optimized method (Table 4).

**Table 4:** CS, TS, and optimized Kano category of each general practice teaching clinic service item.

Items	Traditional category	CS	TS	Optimized category
1	A	0.01	0.83	H (A + M)
2	M	0.02	0.90	H (M + A)
3	A	0.09	0.84	A
4	O	0.01	0.91	H (O + A)
5	I	0.05	0.50	I
6	O	0.09	0.99	O
7	I	0.07	0.50	I
8	A	0.01	0.71	H (A + I)
9	A	0.04	0.70	H (A + I)
10	A	0.11	0.88	A
11	M	0.19	0.97	M
12	O	0.07	0.72	O
13	O	0.15	0.95	O
14	O	0.35	0.98	O

The one-dimensional attributes include “Superior physician provides supplementary consultation,” “Fixed medical team and consistent treatment,” “Reasonable cost for examinations and treatments,” and “Consistent efficacy meets expectations.” When these needs are not met, patients are dissatisfied; when they are met, they are satisfied; and the more they met, the more satisfaction they get. Hospitals should fully satisfy patients’ needs in order to increase their satisfaction and decrease their dissatisfaction.

The attractive attributes include “Advance notice of the specific process and time” and “Health guidance on lifestyle and home care.” These services are surprise feature that exceeds patients’ expectations and greatly enhances their satisfaction. Patients are pleasantly surprised when such needs are met and are not dissatisfied when they are not. Advance notice and more health education are very appealing to patients, and the authors can increase patient satisfaction and bonding by providing these services.

The indifferent attributes include “The resident physician in training leads the treatment process” and “Participation in teacher-student interaction.” These services may not be valued by the patient, or it may be unknown because it have not been exposed to patients, and whether it is provided or not will not affect their satisfaction. This is the main format of our general practice teaching clinic activities, and the results of this survey suggest that patients are not averse to participating in teacher–student interactions during their visits, so we can operate teacher–student interactions, which are also likely to be converted into an attractive need over time.

The must-be attributes include “Skilled in the inspection and operation.” This is a service that patients believe hospitals should provide, and when the need is not met, patients are extremely dissatisfied; and when the need is met, patients are not necessarily satisfied. It is important that we ensure a high level of operational and clinical skills to minimize the level of dissatisfaction and, to some extent, increase the level of patient satisfaction.

The mixed attributes contain five service entries, of which “A comfortable clinic environment” and “Various convenient ways of registration” are a combination of attractive attributes and must-be attributes. They are likely to be developed into must-be attributes in the future and are therefore emphasized to be provided. “Good service attitude” is a mixture of attractive attributes and one-dimensional attributes, and hospitals should not only provide this service but also focus on the quality of that. “Explain the condition and test results in detail and agree on the treatment plan together,” and “Sufficient consultation time (30 min)” are attractive attributes mixed with indifferent attributes. These two services do not become attractive attributes for the time being, but they have the potential to grow into attractive needs in the future, and hospitals should consider the characteristics of attractive needs and undifferentiated needs to determine whether to provide these services.

### SI, DSI and matrix diagram of general practice teaching clinic service demand

As shown in Table 5, it can be seen that the top three highest-ranking items of the Satisfaction Influence values were “Health guidance on lifestyle and home care” (0.76), “Consistent efficacy meets expectations” (0.67), and “Good service attitude” (0.64). The top three highest-ranking services with absolute scores of the Dissatisfied Influence values were “Consistent efficacy meets expectations” (−0.96), “Reasonable cost for examinations and treatments” (−0.90), and “Skilled in the inspection and operation” (−0.88).

**Table 5:** SI and DSI of demand of teaching clinics for patients of general practice.

Items	SI	DSI
1	0.53	−0.52
2	0.56	−0.58
3	0.58	−0.50
4	0.64	−0.59
5	0.48	−0.12
6	0.61	−0.84
7	0.50	−0.14
8	0.50	−0.42
9	0.55	−0.37
10	0.76	−0.45
11	0.44	−0.88
12	0.49	−0.58
13	0.57	−0.90
14	0.67	−0.96

Finally, with the average value of the SI coefficient and the average absolute value of the DSI coefficient (0.5650, −0.5607) as the central point, with the value of the SI coefficient as the horizontal coordinate, and with the value of the DSI coefficient as the longitudinal coordinate, the SI-DSI matrix diagram is obtained, and the service content is divided into four quadrants.

**Figure 1:**
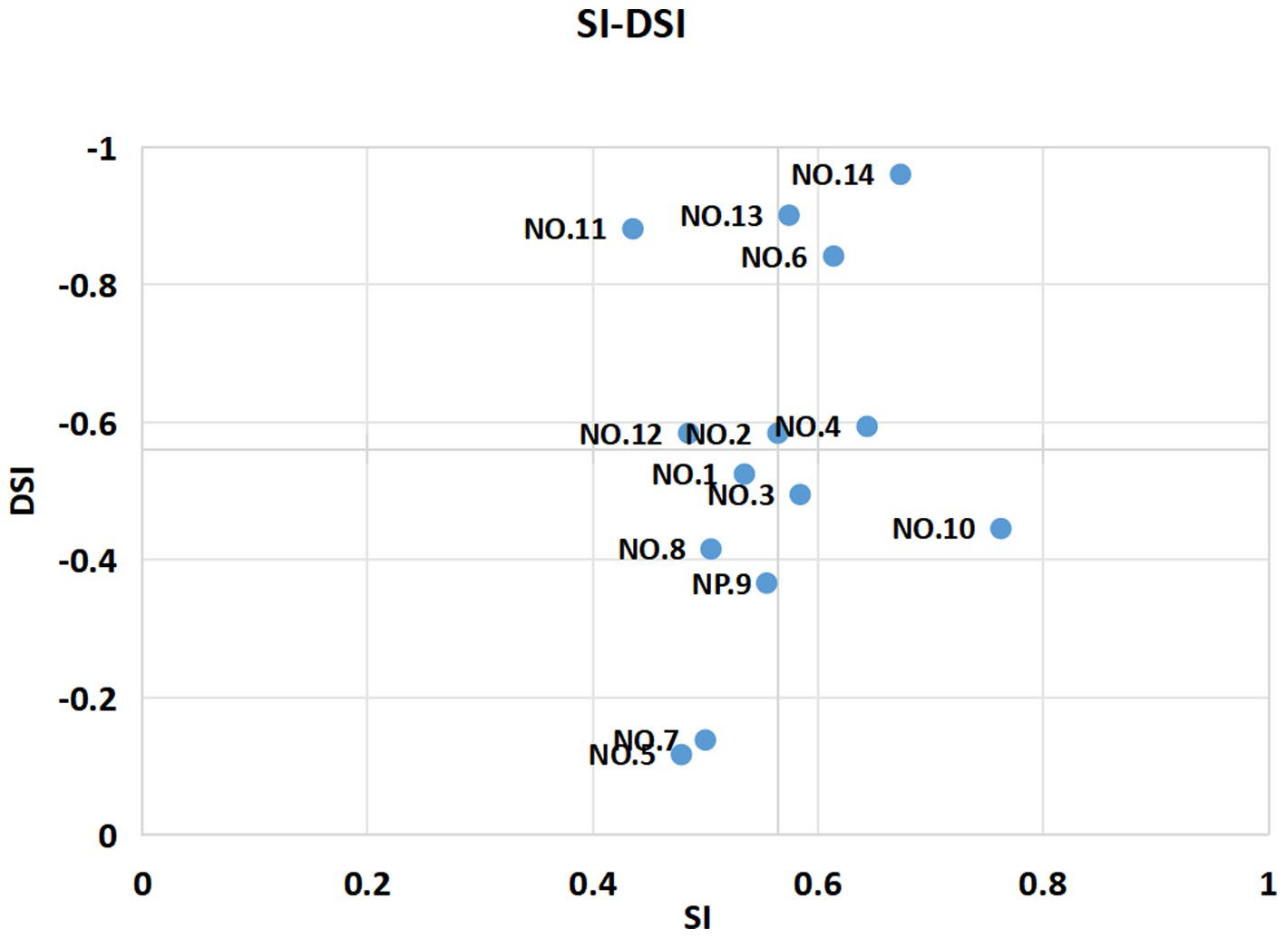
Matrix diagram of general practice teaching clinic service demand.

Figure 1 shows that “Good service attitude,” “Superior physician provides supplementary consultation,” “Reasonable cost for examinations and treatments,” and “Consistent efficacy meets expectations” were one-dimensional attributes. “Various convenient ways of registration,” “Skilled in the inspection and operation,” and “Fixed medical team and consistent treatment” were attractive attributes. “A comfortable clinic environment,” “The resident physician in training leads the treatment process,” “Participate in teacher-student interaction,” “Explain the condition and test results in detail and agree on the treatment plan together,” and “Sufficient consultation time (30 min)” were indifferent attributes. “Advance notice of the specific process and time” and “Health guidance on lifestyle and home care” were must-be attributes.

The SI coefficient and DSI coefficient in the one-dimensional attributes quadrant are both high, which means that patient satisfaction increases significantly after service optimization, and vice versa, the decrease is also obvious. In the attractive attributes quadrant, the SI coefficient is low and the DSI coefficient is high, which means that the improvement of patient satisfaction after service optimization is not obvious, and on the contrary, the decrease is obvious. In the indifferent attributes quadrant, the SI coefficient and DSI coefficient are low, indicating that the improvement of patient satisfaction after service optimization is not obvious, and vice versa. In the must-be attributes quadrant, a high SI coefficient and a low DSI coefficient indicate that patient satisfaction increases significantly after service optimization, and vice versa, the decrease is not obvious. The priority of service items in different quadrants for improvement is as follows: the must-be attributes quadrant, one-dimensional quadrant, attractive attributes quadrant, and indifferent attributes quadrant.

## Discussion

In order to address the issue of “being difficult and expensive to see doctors,” the Chinese government encourages patients to seek an initial diagnosis from primary care providers and has established a two-way referral procedure between primary, secondary, and tertiary care. The General Office of the State Council has announced a goal of increasing the number of general practitioners to five per 10,000 residents by 2030 (20). At present, the training of resident physicians is primarily conducted through ward rotations, focusing more on the diagnosis and treatment of acute and critical cases. This approach may lead to a bias in the spectrum of diseases encountered, making it challenging to gain common and chronic diseases prevalent in the community. In this context, it is difficult for junior general practitioners to establish a holistic view. The future role of general practitioners in China will be centered on community and township health centers. The diagnosis and treatment of common and frequently occurring diseases, as well as the management of chronic diseases, will be the main work of general practitioners. Therefore, the teaching clinic will be an essential part of their training. As early as 1910, medical student teaching clinics were being conducted in North America, and this teaching mode was fully recognized in the Flexner Report (21). China has experienced extensive exploration since the teaching clinic was launched in the General Internal Medicine Department of Peking Union Medical College Hospital in 2008 (22). Although there is still a lack of understanding of teaching clinics in China, many studies have proven that this approach is beneficial for the training of junior doctors and that it not only improves students’ knowledge level, clinical thinking, and independent diagnosis and treatment abilities but also improves their social practice and cultivate good professional spirit (5–7). Research shows that healthcare service quality indicators were the most influential determinants of patient satisfaction (23). Considering the particularity of the general practice teaching clinic mode, it is necessary to understand patients’ acceptance and analyze patients’ demands. Improving the service quality of general practice teaching clinics and patients’ medical experience will contribute to the long-term development of general medicine.

In this study, eight patients expressed a dislike for the teaching clinic approach and did not want to participate in the teacher–student interaction. Among them, seven were below the age of 65, and five had attained high school education or higher. During the investigation, there was a preference for direct treatment by senior doctors in order to expedite the medical treatment process. While most general practice patients did not object to the new clinic approach led by junior doctors, it still necessitated further consultation with senior doctors. The guidance of teachers can help residents win the trust of patients, promote the advancement of teaching activities, and enhance patient adherence. The findings of the study indicated that older patients were more inclined to obtain adequate medical treatment time, medical continuity, and more lifestyle and psychological health education compared to younger patients. This trend may be attributed to the longer duration of illness and the presence of multiple comorbidities among older patients, whereas younger patients are more likely to suffer from a single illness that is more easily managed. It was also found that patients with higher levels of education tended to seek more detailed explanations from doctors regarding their condition and auxiliary examination results. They also expressed a need for consensus between doctors and patients on a diagnosis and treatment plans. In contrast, patients with lower levels of education reported feeling less knowledgeable about medical matters and experienced difficulty in comprehending medical information. Consequently, they primarily relied on doctors for diagnosis and treatment.

The primary purpose of the teaching clinic is to provide training for junior doctors in the diagnosis and treatment of diseases, chronic disease management, doctor–patient communication, and other essential skills. This training aims to deepen junior doctors’ understanding of common clinical issues, health management, and disease screening. Students engage in activities such as reviewing medical history, conducting physical examinations, and formulating medical orders. This experience serves to enhance students’ clinical diagnosis and treatment skills. From the perspective of patients, it is important to provide a clear explanation of the purpose and basic process of teaching clinics in advance. Patients should be informed that, in comparison with traditional clinics, teaching clinics offer longer receiving times and more comprehensive health education and medication guidance services.

The research findings indicate the importance of ensuring that the services provided are “Advance notice of the specific process and time” and “Health guidance on lifestyle and home care.” The service items that are more attractive to patients in a teaching clinic than in a traditional clinic include “Good service attitude,” “Superior physician provides supplementary consultation,” “Reasonable cost for examinations and treatments,” and “Consistent efficacy meets expectations.” Simultaneously, hospitals should focus on providing these services because of their relevance to patients’ experiences. “Various convenient ways of registration,” “Skilled in the inspection and operation,” and “Fixed medical team and consistent treatment” are service programs that can be developed into advantageous programs based on the assurance of meeting the needs of providing must-be attributes service and improving the quality of one-dimensional attributes service, which can be developed into advantageous items, thereby improving the advantages of the teaching clinic and patient cooperation. However, the service attributes outlined in the Kano model are subject to change over time (24), and the initially attractive attributes are gradually adapted, transformed into one-dimensional attributes, and eventually become must-be attributes.

The study has several limitations. First, it is a single-center study. Second, due to the limited duration of teaching outpatient service in the hospital, the patients involved in this study did not take part in the general teaching outpatient service. The next step is to issue questionnaires to patients who have participated in the general teaching clinic and undertake a multi-center study to dynamically track the results of return visits. This will serve as a foundation for enhancing the general teaching clinic.

## Conclusion

In China, the development of general medicine has occurred relatively later compared to other specialized fields. However, there is a significant demand for general practitioners, underscoring the importance of enhancing the training of junior general practitioners. The application of the Kano model for analyzing patient service demand can provide valuable insights into the acceptance and preferences of patients toward general practice teaching clinics. The study found that the majority of patients exhibit a positive attitude toward general practice teaching clinics. This finding offers valuable insights for medical decision-makers to align services with patient needs and supports ongoing enhancements to the development of general practice teaching clinics and the primary medical and health service system.

## Data availability statement

The original contributions presented in the study are included in the article/supplementary material, further inquiries can be directed to the corresponding author.

## Ethics statement

The studies involving humans were approved by the Ethics Committee of the Affiliated Hospital of Yangzhou University (2022-YKL6-04). The studies were conducted in accordance with the local legislation and institutional requirements. The participants provided their written informed consent to participate in this study.

## Author contributions

ZC: Writing – original draft. AY: Writing – review & editing. YW: Writing – review & editing.
